# Persistent, Bioaccumulative, and Toxic Chemicals in Wild Alpine Insects: A Methodological Case Study

**DOI:** 10.1002/etc.5303

**Published:** 2022-03-21

**Authors:** Veronika Rosa Hierlmeier, Nils Struck, Patrick Krapf, Timotheus Kopf, Anna Malena Hofinger, Viktoria Leitner, Philipp Jakob Ernest Stromberger, Korbinian Peter Freier, Florian Michael Steiner, Birgit Christiane Schlick‐Steiner

**Affiliations:** ^1^ Department of Ecology University of Innsbruck Innsbruck Austria; ^2^ Bavarian Environment Agency Garmisch‐Partenkirchen Germany; ^3^ Bavarian Environment Agency Augsburg Germany

**Keywords:** Bioaccumulation, Geometric morphometrics, Insect decline, Mercury, Persistent organic pollutants, Polychlorinated biphenyls

## Abstract

With their high persistence in the environment and their potential for long‐range atmospheric transport, persistent, bioaccumulative, and toxic chemicals (PBTs) may be among the numerous anthropogenic threats to insect populations worldwide. The effects of PBTs on insects have been investigated in the laboratory, but topical field studies are scarce. A reason might be the multiple challenges faced by PBT‐related field studies on wild insects. We studied two species of bumblebees (*Bombus* spp.) and of ants (*Formica* spp.) in two high‐elevation locations in the Austrian and German Alps to tackle two of these challenges. First, PBTs occur in minuscule concentrations compared with other substances in the environment. Therefore, the practicability of body burden data from pooled individuals was tested. Second, fitness proxies like fecundity, which typically are endpoints for chemical toxicity, are difficult to quantify in the field. Hence, fluctuating asymmetry of bumblebee wings and ant heads was tested as an alternative endpoint. To exclude the possibility that fluctuating asymmetry was caused by genetic stressors, inbreeding levels were estimated using population‐genetic markers, and their relationships to fluctuating asymmetry in the same individuals were assessed. We successfully quantified polychlorinated biphenyls and Hg as PBTs using the pooled samples and found PBT data from pooled individuals useful, in that significant correlations to fluctuating asymmetry were identified in bumblebees and ants. This finding confirmed the potential of fluctuating asymmetry to indicate PBT effects in wild insects. Inbreeding did not interfere with PBT links to fluctuating asymmetry in any instance. Our findings contribute to the development of a quantitative methodological framework for investigating the effects of persistent environmental chemicals on wild insects. *Environ Toxicol Chem* 2022;41:1215–1227. © 2022 The Authors. *Environmental Toxicology and Chemistry* published by Wiley Periodicals LLC on behalf of SETAC.

## Graphical abstract

The bioaccumulation of persistent, bioaccumulative, and toxic chemicals in wild alpine insects was proved via chemical analysis. The data showed some significant correlations with measured asymmetries of the body structures of the insects. Inbreeding, analyzed with genetic markers, did not interfere with these links.

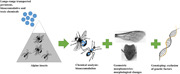

## INTRODUCTION

The importance of insects to the integrity of ecosystems is indisputable. The fact alone that more than half of all known species on earth are insects suggests a high degree of interaction with other organisms and their environment (May, 1992). As primary and secondary consumers, they make up a great part of terrestrial and aquatic food webs. With their enormous biomass in most ecosystems, they facilitate the presence of their numerous predators (Del Toro et al., 2012), and their own feeding is an ecological key driver by itself. The multiplicity of detritivorous and herbivorous insects and their decomposition drive carbon and nutrient cycles, as well as primary production (Belovsky & Slade, 2000, 2018; Speight et al., 2008). In addition, the reproductive success of the majority of flowering plants relies on pollinators (Schoonhoven et al., 2005). Ecosystem integrity may become endangered by a decline in insect diversity and biomass.

Drastic downward trends of insect populations (Eggleton, 2020) and collateral effects on interactions have already been detected. For example, the long‐term decline of insectivorous birds has been linked to insect decline (Møller, 2019; Newton, 2004; Spiller & Dettmers, 2019). In addition, a massive bottom‐up effect due to long‐term decline in arthropods has recently been found in a rainforest food web, which might suggest similar potential consequences regarding insects (Lister & Garcia, 2018). Impacts at the ecosystem level may already be in progress as well. Because the presence or absence of insectivores influences nutrient and carbon cycling, it seems likely that other ongoing factors that reduce insect abundance may affect such ecosystem processes as well (Yang & Gratton, 2014). The decline of insect pollinators has an impact on plant biodiversity, on abundance, and consequently on entire ecosystems, given that an estimated 88% of all flowering plants are pollinated by animals (Ollerton et al., 2011). Highly specialized plants relying on a few pollinator species are especially prone to local extinctions due to insect decline (Biesmeijer et al., 2006). Even disregarding the devastating ecological damage of insect disappearance, the economical and human health consequences could be grave as well. The economic value of insect pollination has been estimated to be at €153 billion for the world's agriculture, and the capacity to feed the world population could be impeded because fruit and vegetable availability is projected to decrease (Gallai et al., 2009).

Habitat loss has been identified as a main factor in insect population decline, but anthropogenic environmental toxicants are other major causes, mainly via agricultural practices, including the application of a broad spectrum of pesticides (Sánchez‐Bayo & Wyckhuys, 2019). However, among the substances that might be responsible for relevant declines of insect populations are, in addition, the persistent organic pollutants (POPs) belonging to persistent, bioaccumulative, and toxic chemicals, the so‐called PBTs (Vallack et al., 1998). Due to their hydrophobic and lipophilic nature, POPs biodegrade slowly in the environment and bioaccumulate along food chains (Jones & de Voogt, 1999). The combination of this persistence with their (semi)volatility allows them to enter the atmosphere and undergo long‐range atmospheric transport (Beyer et al., 2000; Jones & de Voogt, 1999). Even POPs, which have been banned since 2004 by the Stockholm Convention, remain present in the environment due to their high persistence (Hagen & Walls, 2005). Another consequence of the lipophilic nature of POPs is their accumulation in fatty tissue once they have been absorbed by an organism (Jones & de Voogt, 1999). In insects, a fat body serves as storage organ and has various roles related to metabolism and immunity (Azeez et al., 2014). The POPs remain in fat bodies for a long time because the metabolism of fatty tissue tends to be slow. Another prominent effect of POPs is genotoxicity causing, for example, DNA strand breaks (Martínez‐Paz et al., 2013). Phenotypic changes such as alterations in size, shape, or fluctuating asymmetry have also been found to be consequences of high POP loads in vertebrates (Bradley et al., 2019; Jenssen et al., 2010; Romero et al., 2017).

In addition to POPs, the heavy metal mercury (Hg) is a PBT (Buch et al., 2017). It also can bioaccumulate in the tissue of organisms and has toxic effects, but, unlike POPs, Hg cannot be degraded by environmental processes and remains in the ecosystem (Gworek et al., 2020; Jones & de Voogt, 1999; World Health Organisation, 1989). It is transported through the atmosphere long range, and is released from natural sources as well as through human activity (Durnford et al., 2010). It is estimated that the current atmospheric burden of Hg is 4.5 times higher due to atmospheric emissions compared with the natural background (Arctic Monitoring and Assessment Programme, Norway/UN Environment Programme, Chemicals and Health Branch, 2019). To reduce anthropogenic emissions of Hg and Hg compounds, an international agreement, the Minamata Convention, came into force in 2017. Nevertheless, Hg will remain problematic over decades. Sedimentation is the only global sink, and due to its high volatility, Hg is circulating to a large degree among the atmosphere, biosphere, hydrosphere, and pedosphere (Gworek et al., 2020; Saito, 2019).

Various pollutants induce fluctuating asymmetry through developmental disturbance in wild vertebrates at low concentrations (Lajus et al., 2015; Montalvão et al., 2018; Yalkovskaya et al., 2016; Zhelev et al., 2015). Honeybees have also been found to exhibit increased fluctuating asymmetry due to exposure to insecticides (Ondo Zue Abaga et al., 2011). Because of these findings, a direct impact of PBTs on insect fluctuating asymmetry seems likely. Particularly high pollutant loads can be found in mountainous regions of temperate zones due to high levels of precipitation combined with their proximity to major pollutant sources (Blais et al., 1998). Nevertheless, our knowledge of the impact of PBTs on high‐elevation ecosystems is limited (Bizzotto et al., 2009; Miner et al., 2017; Pittino et al., 2018). In fact, much of the PBT toxicity‐related research is based on laboratory studies, whereas field studies on wild insects address either bioaccumulation, bioamplification, or dispersal of pollutants within or into trophic chains, often in aquatic ecosystems (Bartrons et al., 2012; Bizzotto et al., 2009; Daley et al., 2011; Mommaerts et al., 2011; Raikow et al., 2011; Singh, 2013). Moreover, there is a lack of studies measuring direct effects of PBTs (e.g., morphological changes) on wild insects in a noncontrolled environment. Reasons for this lack might be, among others, the challenges inherent in such field studies. To the best of our knowledge, our study is the first field study to examine potential toxicological effects of PBTs on terrestrial insects in a high‐elevation ecosystem.

In the present study, we investigated practical solutions to the challenges of PBT research in wild insects, and we sketch a methodological framework for future studies. First, PBT concentrations in the field are unknown a priori. When researchers are working with insects, the PBT concentrations of individuals will be below the detection threshold for a proper chemical analysis due to their small size. To obtain a sufficient biomass for chemical analysis, it is common to use pooled samples of insects, meaning that differences can only be detected across pools, for example, from different sampling locations (Hare et al., 1991). The chemical analysis of single individuals, to link effects of PBTs in detail, is a methodological challenge. Second, fitness proxies, like fecundity or life history traits, are hard to examine in a noncontrolled environment. Fluctuating asymmetry is a well‐established measure of developmental instability and is also a suitable stress indicator (Møller & Swaddle, 1997). Because PBTs are known stressors in both humans and wildlife, a correlation of fluctuating asymmetry and PBT body burden can be envisaged. Finding evidence for a link between morphological changes and the concentration of PBTs in organisms may encourage further research on PBTs in the field. Other environmental chemicals can also trigger fluctuating asymmetry (Arambourou et al., 2012). In our study, polychlorinated biphenyls (PCBs) serve as a proxy. Potentially detected fluctuating asymmetries can thus be due to PCBs and/or other co‐occuring substances. Fluctuating asymmetry, used as an indicator of the impact of PBTs on individuals, could also be influenced by endogenous genetic factors such as inbreeding and/or physical causes, which could cause developmental instabilities (Băncilǎ et al., 2010; Bjorksten et al., 2001).

To address these two challenges, we conducted a pilot study using bumblebees and ants sampled at two high‐elevation locations. We tested the practicability of pooled samples examining PCBs and Hg. These substances are widely distributed in the environment, and analytical methods for the detection in biota are well established (Jones & de Voogt, 1999). To measure PBT‐induced stress in wild individuals, fluctuating asymmetry was tested as an indicator. To screen for a potential influence on fluctuating asymmetry by inbreeding, population‐genetic analyses using microsatellite loci were done. We propose that the present study is a methodological blueprint for a research topic that urgently requires the attention of the scientific community.

## MATERIALS AND METHODS

### Study sites and organisms

*Bombus* spp. and *Formica* spp. were sampled in alpine habitats on Zugspitze in Germany and on Hoher Sonnblick in Austria. At both locations, concentrations of PCBs, Hg, organochlorine pesticides (OCPs), polycyclic aromatic hydrocarbons, halogenated flame retardants, and chlorinated dioxins and furans (PCDD/F) in ambient air and bulk deposition have been continuously monitored since 2005 by the Environmental Research Station Schneefernerhaus on Zugspitze and by the Sonnblick Observatory at Hoher Sonnblick (Umweltbundesamt & Bayerisches Landesamt für Umwelt, 2020). Detailed information on the study sites can be found in Jakobi et al. (2015). The sampling areas of insects were located in the surroundings of the Schneefernerhaus and the Sonnblick Observatory at an altitude of 1600–1900 m above sea level, an area dominated by shrub and heath vegetation.

To analyze PCBs and Hg, an insect biomass of 3.5 g would be sufficient, but to analyze a broad spectrum of PBTs in further studies, a total amount of 40 g (minimum value, Umweltbundesamt) insect biomass was sampled. To achieve this biomass, either relatively big or very abundant study organisms were needed. We chose bumblebees (*Bombus* spp.) and wood ants (*Formica* spp.) as target organisms. Studies applying geometric morphometrics of ants and bumblebees had also already been conducted (Aytekin et al., 2007; Wagner et al., 2017). Because the *Bombus* species composition at the sampling sites was not known at the beginning of the present study, all bumblebees were collected for later identification. Bumblebees were not collected in nests, to keep the pressure on single colonies low. In contrast, a small fraction of the workers in the wood ant nests was sampled, because nests contain very high numbers of individuals (Pisarski, 1982; Rosengren et al., 1987).

### Field work

Sampling was done in August and September 2018. Bumblebees were individually caught using Duran® borosilicate glass flasks with ground glass joints (100 ml). Ants were collected using forceps, but also with a motorized suction device to avoid contact with formic acid. Flasks with bumblebees and ants were intermediately stored in a cool bag followed by euthanizing without contamination by inserting the flasks in a dry shipper filled with liquid nitrogen (−196 °C).

Due to possible alteration in PBT concentrations when insects are stored in ethanol, the use of ethanol was avoided. Instead, the sampled insects were wrapped in aluminum foil on arrival in the laboratory, transferred into glass tubes, and stored in a freezer at −20 °C. This guaranteed a safe conservation of DNA without altering PBT body loads. To avoid the contamination of samples, all utensils that came in contact with the sampled insects were cleaned using a cleaning protocol with sequential usage of toluene, acetone, water/detergent, and finally isohexane Picograde® (LGC Standards) and dry heat (Supporting Information, Table S1).

### Species identification

Bumblebees and ants were morphologically identified at the species level using a Nikon SMZ‐U stereo microscope (maximal magnification: 80×) and the identification keys by Amiet et al. (2017) and Seifert (2018) for bumblebees and ants, respectively. To avoid contamination, the samples were identified on aluminum foil as underlay. Most of the bumblebees belonged to the *Bombus lucorum* complex, namely, the morphologically hardly distinguishable species *B. lucorum* and *Bombus cryptarum* (Bossert, 2015). For the chemical analyses, all bumblebees collected were used (Table 1), but for the morphological and genetic analyses, just the individuals of the *B. lucorum* complex (Table 2; for an additional, graphical visualization, see the Supporting Information, Figure S1). Using cytochrome c oxidase I (COI) sequencing, individuals of the *B. lucorum* complex and individuals of *Formica sensu stricto* were identified at the species level.

**Table 1:** Location, species, number of individuals, and biomass/pooled sample for chemical analyses

Sample code	Location	Species	No. of individuals	Biomass (g)
Pool 1	Zugspitze	*Bombus* spp.	250	39.8
Pool 2	Zugspitze	*Formica aquilonia*	5000 (estimated)	40.0
Pool 3	Zugspitze	*Formica exsecta*	800 (estimated)	4.9
Pool 4	Hoher Sonnblick	*Bombus* spp.	96	15.5
Pool 5	Hoher Sonnblick	*F. aquilonia*	5000 (estimated)	40.0
Pool 6	Hoher Sonnblick	*F. exsecta*	2600 (estimated)	15.7

**Table 2:** Number of specimens/genera, species, and location^a^

Location	*Bombus cryptarum*	*Bombus lucorum*	*Bombus* indet.	*Formica aquilonia*	*Formica exsecta*
Zugspitze	31	31	2	67 (7)	49 (5)
Hoher Sonnblick	1	2	1	48 (5)	40 (4)
Total	32	33	3	115 (12)	89 (9)

^a^Only individuals included in fluctuating asymmetry and microsatellite analyses shown. Number of ant nests are shown in parenthesis.

indet. = unidentified species.

For COI sequencing, DNA was extracted using the QIAGEN DNeasy® Blood & Tissue Kit (Qiagen) after homogenizing one bumblebee leg or ant body (minus the head). For ants, one individual was used/nest. Per individual, 70 µl were eluted using distilled water. Polymerase chain reaction (PCR) was done in 1× reaction buffer, 0.2 μM of each primer, 0.25 U MyTaq polymerase (Bioline), and approximately 50 ng template DNA in a total volume of 5 μl. The presence of amplicon was checked by agarose gel electrophoresis. The PCR products were purified by adding 2 U Exonuclease I (Thermo Scientific) and 0.1 U FastAP (Thermo Scientific), followed by two 15‐min incubations at 37 and 80 °C, respectively. Sanger sequencing was done by a commercial provider (Eurofins MWG Operon) using the forward and reverse amplification primer. The primers for bumblebees (F: 5′‐ATAATTTTTTTTATAGTTATA‐3′; R: 5′‐GATATTAATCCTAAAAAATGTTGAGG‐3′) were originally developed for *Apis mellifera* by Tanaka et al. (2001) and were used to identify the species of the *B. lucorum* complex by Murray et al. (2008). For ants, we used the universal COI primers LCO1490 and HCO2198 (5′‐GGTCAACAAATCATAAAGATATTGG‐3′ and 5′‐TAAACTTCAGGGTGACCAAAAAAT CA‐3′ respectively; Supporting Information, Table S2; Folmer et al., 1994). A neighbor‐joining tree based on genetic distance was compiled using Mega Ver 6. Two clades were found and all individuals were aligned with sequences (accession numbers: AY530010 and AY530012) stored in GenBank using BLAST. Ant sequences were also aligned with sequences (accession number: KX665008) stored in GenBank using BLAST.

### Geometric morphometrics

**Figure 1:**
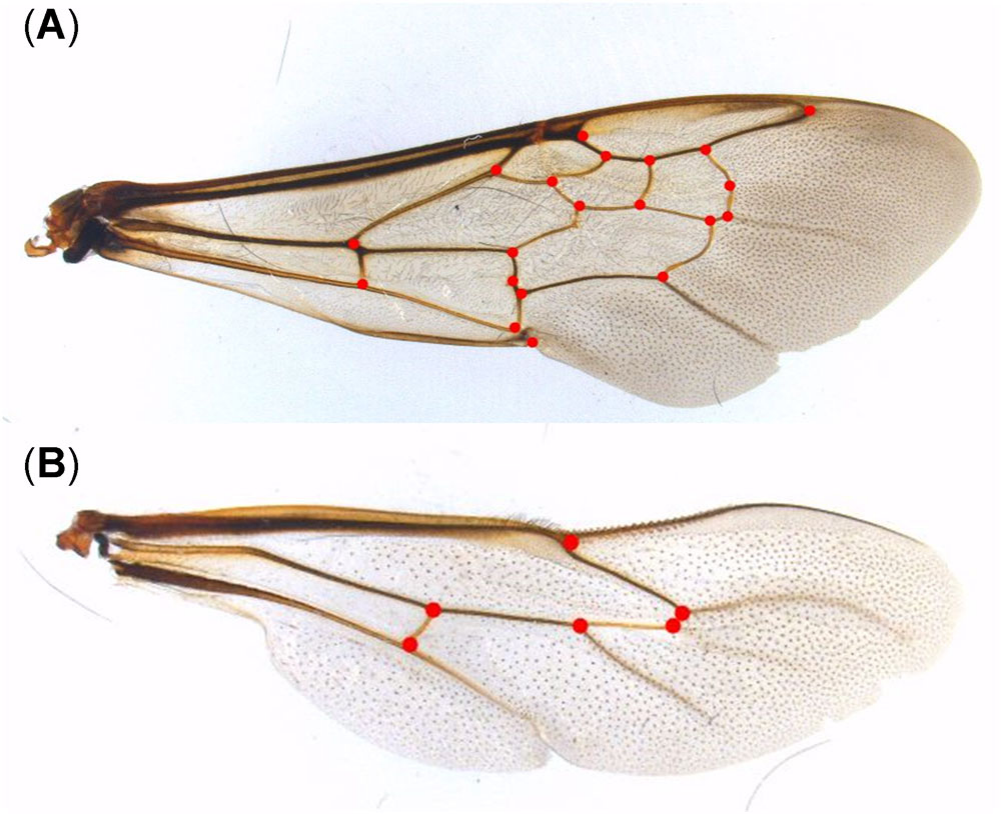
Morphological parameters used to evaluate fluctuating asymmetry of wings of *Bombus lucorum* complex. Red dots indicate the position of the 20 and 6 landmarks used on detached forewing (**A**) and hindwing (**B**), respectively. Landmarks were positioned at the nadirs of the distal contour lines of vein intersections. Thus, distances could be measured on the wings, and changes in asymmetry could be determined.

**Figure 2:**
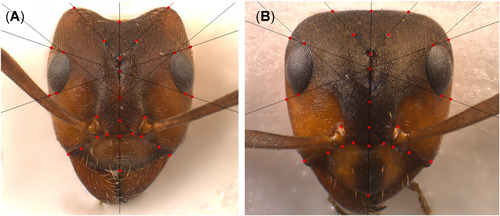
Morphological parameters used to evaluate fluctuating asymmetry of heads. Red dots indicate the position of the 29 landmarks used on photographed heads of *Formica exsecta* (**A**) and *Formica aquilonia* (**B**). Landmarks were positioned with the help of auxiliary lines on distinctive points on the heads. Thus, distances could be measured on the left and right side of an individual's head, and changes in asymmetry could be determined.

Geometric morphometrics of bumblebees was done using photographs of detached fore‐ and hindwings of females. Ten ant heads/nest (chosen to represent size variation in the nest) were prepared on needles, and photographs of the frontal view were taken. All photographs were taken using a Leica Z6 APO macroscope with a Leica DFC 420 camera and processed with LAS Ver 3.6.0. The .tps files were built from these images using tpsUtil v1.76 (Rohlf, 2015). Then tpsUtil and Notepad++ Ver 7.8.2 were used to manipulate and prepare the.tps files for analysis. Landmarks were set using tpsDig2 Ver 2.31 (Rohlf, 2015). On fore‐ and hindwings, 20 and 6 landmarks were placed, respectively (Figure 1). The positions of these landmarks were taken from Aytekin et al. (2007). Twenty‐nine landmarks were set on ant heads (Figure 2).

Generating empirical evidence for landmarking consistency is key to guarantee reliable results in geometric morphometrics. Landmark locations of bumblebee wings were defined at a corner of a vein crossing instead of in the middle of the crossing. By placing the landmarks consistently at the nadirs of the distal contour lines of vein intersections rather than placing them on the centroids of intersections, the reproducibility of the data was increased (pilot study, data not shown). For ants, auxiliary lines were used to determine the exact points for the landmarks. To quantify the digitizing error, 20 bumblebees and 40 ants from one sampling location were digitized a second time. For these bumblebees, eight photographs were taken per individual, from both sides of each of the four wings, to quantify a potential imaging error. The ants were also photographed a second time to include the imaging error. For bumblebees, a Procrustes analysis of variance (analysis of variance [ANOVA], *α* = 0.05) was done comparing individuals, left and right wings, both wing sides (imaging error), and first and second digitizing run (digitizing error). For ants, a similar Procrustes ANOVA (*α* = 0.05) was done to compare individuals, nests, first and second photographs (imaging error), and first and second digitizing run to quantify the digitizing error (Wagner et al., 2017).

Fluctuating asymmetry was analyzed using MorphoJ Ver 1.07a (Klingenberg, 2011). Classifiers (“individual,” “side,” “updown” for bumblebees; “nest,” “mountain” for ants) were determined. A Procrustes fit was done to properly arrange and reposition the landmarks of each individual photo. A Procrustes ANOVA (*α* = 0.05) was done to compare individual specimens, left and right sides (i.e., bumblebee wings or left and right halves of ant heads). The imaging error was included as a reference for the variation explained. Finally, the resulting data were extracted and edited using Microsoft Excel Ver 1908. The fluctuating asymmetry index 2 (FA2) of wing and head shape was calculated for each individual following Palmer and Strobeck (2003): (*R* − L)/([*R* + *L*]/2), in which *R* and *L* are defined as the size of the right and left side of the individual.

### Microsatellite genotyping and population genetics

Only diploid worker ants were used in the present study. Genetic analyses were conducted exclusively on female bumblebees. The DNA extraction for the microsatellite analysis followed the same protocol as described in the section Species identification. The individuals that underwent microsatellite analysis were the same that were photographed for geometric morphometrics. Bumblebees were genotyped at seven microsatellite loci (198, 327, 601, BT04, BT10, BT23, and BL13) developed for species of the *B. lucorum* complex (Reber Funk et al., 2006; Stolle et al., 2011). Each bumblebee sample of 5 µl contained a master mix including 0.5 µl of sample DNA, distilled water 5× OneTaq® Quick‐Load® Reaction Buffer, 10 mM dNTPs, 10 µM M13‐tailed forward primer, 10 µM untailed reverse primer, 10 µM fluorescent labeled M13 primer, and OneTaq® Quick‐Load® DNA Polymerase (New England Biolabs).

The PCR of ant samples was done using two methods, depending on the loci. For the loci Fy3, FE16, FE17, FE37, FE38, and FE51 (Gyllenstrand et al., 2002; Hasegawa & Imai, 2004), a 5‐µl master mix was prepared including 0.5 µl of sample DNA, distilled water, 2× Rotor‐Gene Master Mix (Qiagen), 0.02 μM M13‐tailed forward primer, 0.2 μM fluorescent labeled M13 primer, and 0.2 μM untailed reverse primer. For the remaining loci, FL21, FE7, FE11, FE13, FE15, FE42, FE49, and P22 (Chapuisat, 1996; Gyllenstrand et al., 2002; Trontti et al., 2003), a 5‐µl master mix was prepared including 0.5 µl of sample DNA, H_2_O distilled water, 5× OneTaq® Quick‐Load® Reaction Buffer (New England Biolabs), 10 mM dNTPs, 10 µM M13‐tailed forward primer, 10 µM untailed reverse primer, 10 µM fluorescent labeled M13 primer, and OneTaq® Quick‐Load® DNA Polymerase (New England Biolabs; Supporting Information, Tables S3 and S4). The PCRs were carried out on a UnoCycler (VWR). Fragment analysis for bumblebees and ants was carried out on an ABI3730XL genetic analyzer (Applied Biosystems) by a commercial provider (Comprehensive Cancer Center DNA Sequencing & Genotyping Facility, University of Chicago, USA). Traces were visualized using PeakScanner software Ver 2.0 (Applied Biosystems) and scored manually. To verify the adequacy of the selected mitochondrial markers, linkage disequilibrium and Hardy–Weinberg equilibrium deviation were tested using Genepop Ver 4.2 (Raymond & Rousset, 1995; Rousset, 2008), and a Bonferroni–Holm correction for multiple testing was applied. Multilocus heterozygosity and mean squared distance between alleles (*d*²) were calculated manually based on microsatellite alleles to quantify inbreeding in bumblebee and ant populations (Coulson et al., 1998; Hansson, 2010). Statistical differences of inbreeding indices between species within locations and between locations within species were analyzed via two‐sample *t*‐tests or Mann–Whitney *U* tests depending on whether the data were normally distributed or not, respectively. The significance level was set at *α* = 0.05 and was correspondingly lower in the case of correcting for multiple comparison. All tests comparing means were run using R and Bonferroni–Holm corrected for multiple testing. In addition, expected and observed heterozygosity (*H*_e_ and *H*_o_, respectively) were analyzed using Genepop Ver 4.2.

### Chemical analysis of body burdens

For the chemical analyses, worker ants were pooled according to species and study sites to obtain enough biomass. This resulted in a total of four pooled samples, consisting of one pooled sample of individuals of *Formica exsecta* and one pooled sample of individuals of *Formica aquilonia* for each study site (Table 1). For the same reason, bumblebees of both sexes of the *B. lucorum* complex and nine other bumblebee species (*Bombus jonellus*, *Bombus monticola*, *Bombus pratorum*, *Protachorutes pyrenaeus*, *Bombus rupestris*, *Bombus sichelii*, *Bombus soroeensis*, *Bombus sylvestris*, and *Bombus wurflenii*) were pooled for the chemical analysis, thus forming one pool/site. The remaining bumblebees needed to be sampled to obtain enough biomass in the late sampling season. In total, there were two pooled samples of bumblebees, one/study site (Table 1). The PBT concentrations were measured by the accredited laboratory of the Environment Agency Austria in Vienna. Prior to the analysis, the samples were homogenized and lyophilized. Isotope‐labeled standards for the investigated PCBs were added to the samples for quality control, and repeated measurements by the laboratory show that a deviation of 30% is to be expected as standard (Eppe et al., 2015). For Hg, a deviation of 13% is to be assumed (Umweltbundesamt, 2015). The Hg and PCB concentrations were determined via atomic fluorescence spectroscopy and gas chromatography/high‐resolution mass spectrometry, respectively. The PCBs we investigated are specified by the United Nations Environment Programme (2013) as indicator PCBs for monitoring studies: PCB 28 (2,4,4′‐trichlorobiphenyl), PCB 52 (2,2′,5,5′‐tetrachlorobiphenyl), PCB 101 (2,2′,4,5,5′‐pentachlorobiphenyl), PCB 138 (2,2′,3,4,4′,5′‐hexachlorobiphenyl), PCB 153 (2,2′,4,4′,5,5′‐hexachlorobiphenyl), and PCB 180 (2,2′,3,4,4′,5,5′‐heptachlorobiphenyl). These indicator PCBs are widely used as a proxy for total PCB quantity due to their wide range in chlorine numbers and their strong presence in most environments (Lang, 1992).

### Statistical evaluation of PBT–fluctuating asymmetry–inbreeding links

To detect potential effects of PBT concentrations or inbreeding on the organisms, linear models were fit using R Ver 3.6.3 and RStudio Ver 1.2.1335, using the base stats R‐package. In the models using bumblebees, the independent variables were the body burden data of the pooled samples (Pool 1 and Pool 4). The FA2 values of either front or hind wings and inbreeding coefficients of the 64 individuals of the *B. lucorum complex* out of Pool 1 and the four individuals of the *B. lucorum* complex out of Pool 4 were set as dependent variables. Due to the restricted sample size on Hoher Sonnblick with only four data points, both sampling locations were grouped together and not included as fixed effect in the models.

In the models using ants, the independent variables were represented by the pooled body burden data of Pools 2, 3, 5, and 6. The FA2 values of the head and the inbreeding coefficients of 67 individuals of Pool 2, 49 individuals of Pool 3, 48 individuals of Pool 5, and 40 individuals of Pool 6 were set as dependent variable. For bumblebees, this equaled 28 calculations (two fluctuating asymmetry index values, two heterozygosity measures, seven PBT concentrations) and for ants 14 calculations (one fluctuating asymmetry index value, two heterozygosity measures, seven PBT concentrations). Model fits were checked via scaled residual plots and Kolmogorov–Smirnov tests (*α* = 0.05) using the R‐package DHARMa Ver 0.3.1 (Hartig, 2020). Some models using the fluctuating asymmetry of the bumblebee forewing and hindwing as a dependent variable revealed a poor model fit. A Tukey's ladder of power was applied on these variables using the function “transformTukey” in the package rcompanion (Mangiafico, 2020) to produce more normally distributed values. For the fluctuating asymmetry of forewing and hindwing, the selected lambdas for the data transformation were −1.15 and −0.3, respectively, and yielded an improved model fit.

## RESULTS

### Sample size and species composition

In total, 46 females and 50 males of bumblebees were collected at Hoher Sonnblick, resulting in 15.5 g of biomass for chemical analysis (Table 1). Four females belonged to the *B. lucorum* complex (Table 2). At Zugspitze, 103 females and 147 males with a biomass of 39.8 g were collected. Sixty‐four females belonged to the *B. lucorum* complex. In total, two individuals could not be identified at the species level. Two ant species were identified morphologically and via COI sequencing: *F. aquilonia* and *F. exsecta*. At Hoher Sonnblick, a total of 48 individuals of *F. aquilonia* from five nests and a total of 40 individuals of *F. exsecta* from four nests were collected each for genetic and morphological analysis (Table 2). More individuals of *F. aquilonia* and *F. exsecta* (not counted but estimated at 5000 and 2600 workers, respectively) were collected for chemical analysis, resulting in 40.0 and 15.7 g, respectively (Table 1). At Zugspitze, a total of 67 individuals of *F. aquilonia* from seven nests and 49 individuals of *F. exsecta* from five nests were collected for genetic and morphological analysis. For chemical analysis, 40 g of *F. aquilonia* and 4.9 g of *F. exsecta* were collected in addition (individuals were not counted but estimated at 5000 and 800 workers, respectively, Table 1).

### Fluctuating asymmetry

The Procrustes ANOVA for the landmarking validation revealed statistically nonsignificant imaging and digitizing errors for bumblebee and ant landmarking. Regarding the mean squares and *F*‐values of bumblebees, the variation explained by individual differences and fluctuating asymmetry was higher than the imaging error. In ants, a similar pattern was noticeable: Both mean squares and *F*‐values of the individual and nest comparisons were higher than the two errors (Supporting Information, Table S5).

The Procrustes ANOVA yielded consistent results for the overall fluctuating asymmetry of bumblebee wings (Table 3). In each analysis, the explained variation was highest among individuals, followed by fluctuating asymmetry between left and right wings (both significant) of the individuals. For the forewings of the Zugspitze individuals, the *F*‐values remained higher than 1, and combined with the low *p*‐value, this indicated a significant fluctuating asymmetry in these individuals. The Procrustes ANOVA also revealed consistent results for the overall fluctuating asymmetry of ants (Table 4). Significant differences were found between both locations in both species. The difference between locations was expressed more strongly in *F. exsecta* than in *F. aquilonia* when the explained variation was compared. It also explained more variation than the fluctuating asymmetrys of left and right head sides in both species. The fluctuating asymmetry always explained less variation than locations or individuals.

**Table 3:** Results of the Procrustes analysis of variance comparing left and right fore‐ and hindwings of bumblebees from both locations

Object	Effect	Sum of squares	Mean squares	*df*	*F*	*p*
Zugspitze forewings	Individual	1.22 × 10^−1^	5.66 × 10^−5^	2.16 × 10^3^	3.66	<0.0001
	Fluctuating asymmetry	3.34 × 10^−2^	1.55 × 10^−5^	2.16 × 10^3^	1.41	<0.0001
Zugspitze hindwings	Individual	1.26 × 10^−1^	2.58 × 10^−4^	488	7.43	<0.0001
	Fluctuating asymmetry	1.69 × 10^−2^	3.47 × 10^−5^	488	3.81	<0.0001
Hoher Sonnblick forewings	Individual	5.75 × 10^−3^	5.32 × 10^−5^	108	13.6	<0.0001
	Fluctuating asymmetry	4.22 × 10^−4^	3.91 × 10^−6^	108	4.28	<0.0001
Hoher Sonnblick hindwings	Individual	6.97 × 10^−3^	2.90 × 10^−4^	24	10.6	<0.0001
	Fluctuating asymmetry	6.57 × 10^−4^	2.74 × 10^−5^	24	3.18	0.0001

**Table 4:** Results of the Procrustes analysis of variance comparing left and right sides of ant heads from both locations^a^

Object	Effect	Sum of squares	Mean squares	*df*	*F*	*p*
*Formica aquilonia*	Location	3.20 × 10^−3^	1.19 × 10^−4^	27	3.96	<0.0001
	Individual	9.14 × 10^−2^	3.00 × 10^−5^	3.05 × 10^3^	6.00	<0.0001
	Fluctuating asymmetry	1.54 × 10^−2^	4.99 × 10^−6^	3.08 × 10^3^	—	—
*Formica exsecta*	Location	7.16 × 10^−3^	2.65 × 10^−4^	27	9.72	<0.0001
	Individual	6.40 × 10^−2^	2.73 × 10^5^	2.35 × 10^3^	4.32	<0.0001
	Fluctuating asymmetry	1.50 × 10^−2^	6.31 × 10^6^	2.38 × 10^3^	—	—

^a^Because of the object symmetry (bilateral) of the ant heads, no *F* and *p*‐value could be calculated (second landmark setting and comparison of both analysis necessary).

### Microsatellite analysis

In *B. cryptarum*, 11 of 21 locus combinations were significantly linked. and 4 of 7 loci deviated significantly from the Hardy–Weinberg equilibrium after Bonferroni–Holm correction. One of the seven loci depicted a higher *H*_o_ than *H*_e_ (Supporting Information, Table S6). In *B. lucorum*, no locus combination was significantly linked, and one of the seven loci deviated significantly from the Hardy–Weinberg equilibrium after Bonferroni–Holm correction. Two of the seven loci depicted a higher *H*_o_ than *H*_e_. The Mann–Whitney *U* test comparing both bumblebee species on Zugspitze showed a significant difference when comparing multilocus heterozygosity (*W* = 235.5, *p* = 0.007), with *B. lucorum* depicting a significantly higher heterozygosity mean (0.7871) than *B. cryptarum* (0.6270). No difference in *d* ^2^ was found (Mann–Whitney *U* test, *W* = 452, *p* = 0.457). The Mann–Whitney *U* tests comparing both ant species from Zugspitze yielded no significant differences, for either multilocus heterozygosity or *d*² (*W* = 1589.5, *p* = 0.773 and *W* = 1526.5, *p* = 0.522, respectively). The inbreeding values were never significantly linked to fluctuating asymmetry of forewings and hindwings (Table 5).

**Table 5:** Linear models for effects of inbreeding values and PBT measurements on shape of bumblebee fore‐ and hindwings and linear models for effects of inbreeding values, PBT measurements, and location on ant head shape

Organism	Model (z ∼ ax + by + c)	*t*1	*t*2	*t*3	*R* ^2^
*Bombus* spp.	Fore shape FA ~ MLH + Hg	0.720	3.86***	n.a.	0.19***
	Fore shape FA ~ MLH + PCB 28	0.720	−3.86***	n.a.	0.19***
	Fore shape FA ~ MLH + PCB 52	0.720	−3.86***	n.a.	0.19***
	Fore shape FA ~ MLH + PCB 101	0.720	−3.86***	n.a.	0.19***
	Fore shape FA ~ MLH + PCB 138	0.720	3.86***	n.a.	0.19***
	Fore shape FA ~ MLH + PCB 153	0.720	3.86***	n.a.	0.19***
	Fore shape FA ~ MLH + PCB 180	0.720	3.86***	n.a.	0.19***
	Fore shape FA ~ *d* ^2^ + Hg	0.241	3.95***	n.a.	0.18***
	Fore shape FA ~ *d* ^2^ + PCB 28	0.241	−3.95***	n.a.	0.18***
	Fore shape FA ~ *d* ^2^ + PCB 52	0.241	−3.95***	n.a.	0.18***
	Fore shape FA ~ *d* ^2^ + PCB 101	0.241	−3.95***	n.a.	0.18***
	Fore shape FA ~ *d* ^2^ + PCB 138	0.241	3.95***	n.a.	0.18***
	Fore shape FA ~ *d* ^2^ + PCB 153	0.241	3.95***	n.a.	0.18***
	Fore shape FA ~ *d* ^2^ + PCB 180	0.241	3.95***	n.a.	0.18***
	Hind shape FA ~ MLH + Hg	0.153	1.29	n.a.	−0.01
	Hind shape FA ~ MLH + PCB 28	0.153	−1.29	n.a.	−0.01
	Hind shape FA ~ MLH + PCB 52	0.153	−1.29	n.a.	−0.01
	Hind shape FA ~ MLH + PCB 101	0.153	−1.29	n.a.	−0.01
	Hind shape FA ~ MLH + PCB 138	0.153	1.29	n.a.	−0.01
	Hind shape FA ~ MLH + PCB 153	0.153	1.29	n.a.	−0.01
	Hind shape FA ~ MLH + PCB 180	0.153	1.29	n.a.	−0.01
	Hind shape FA ~ *d* ^2^ + Hg	0.045	−1.32	n.a.	−0.01
	Hind shape FA ~ *d* ^2^ + PCB 28	0.045	−1.32	n.a.	−0.01
	Hind shape FA ~ *d* ^2^ + PCB 52	0.045	−1.32	n.a.	−0.01
	Hind shape FA ~ *d* ^2^ + PCB 101	0.045	−1.32	n.a.	−0.01
	Hind shape FA ~ *d* ^2^ + PCB 138	0.045	1.32	n.a.	−0.01
	Hind shape FA ~ *d* ^2^ + PCB 153	0.045	1.32	n.a.	−0.01
	Hind shape FA ~ *d* ^2^ + PCB 180	0.045	1.32	n.a.	−0.01
*Formica* spp.	Head shape FA ~ MLH + Hg + location	−0.998	−1.97	−2.52*	0.03*
	Head shape FA ~ MLH + PCB 28 + location	−1.20	3.21**	−2.12*	0.06**
	Head shape FA ~ MLH + PCB 52 + location	−1.20	3.24**	−2.74**	0.06**
	Head shape FA ~ MLH + PCB 101 + location	−1.20	3.23**	−3.79***	0.06**
	Head shape FA ~ MLH + PCB 138 + location	−1.15	−2.64**	−3.27**	0.06**
	Head shape FA ~ MLH + PCB 153 + location	−0.630	−0.46	−1.53	0.01
	Head shape FA ~ MLH + PCB 180 + location	−0.457	0.65	−1.26	0.01
	Head shape FA ~ *d* ^2^ + Hg + location	−1.065	−1.61	−2.28*	0.03
	Head shape FA ~ *d* ^2^ + PCB 28 + location	−0.800	2.83**	−1.94	0.05**
	Head shape FA ~ *d* ^2^ + PCB 52 + location	−0.794	2.86**	−2.50*	0.05**
	Head shape FA ~ *d* ^2^ + PCB 101 + location	−0.796	2.85**	−3.42***	0.05**
	Head shape FA ~ *d* ^2^ + PCB 138 + location	−0.924	−2.25*	−2.91**	0.05**
	Head shape FA ~ *d* ^2^ + PCB 153 + location	−1.27	−0.25	−1.30	0.01
	Head shape FA ~ *d* ^2^ + PCB 180 + location	−1.31	0.74	−1.13	0.02

* *p* < 0.05.

** *p* < 0.01.

*** *p* < 0.001.

PBT = persistent, bioaccumulative, and toxic chemical; Hg = mercury; PCB = polychlorinated biphenyl; *d* ^2^ = mean squared distance between alleles; FA = fluctuating asymmetry; MLH = multilocus heterozygosity; *R* ^2^ = coefficient of determination of the linear model; *t*1 = *t*‐value of independent variable 1 (inbreeding values); *t*2 = *t*‐value of independent variable 2 (PBTs); *t*3 = I‐value of independent variable 3 (location); n.a. = data not available.

In *F. aquilonia*, 11 of the 156 locus combinations were significantly linked, and 8 and 6 of the 13 loci deviated significantly from the Hardy–Weinberg equilibrium after Bonferroni–Holm correction on Zugspitze and Hoher Sonnblick, respectively. Four of the 13 loci depicted a higher *H*_o_ than *H*_e_ on both Zugspitze and Hoher Sonnblick (Supporting Information, Table S7). In *F. exsecta*, 85 of 132 locus combinations were significantly linked, and 7 of 12 loci deviated significantly from the Hardy–Weinberg equilibrium after Bonferroni–Holm correction in both locations. Eight of 12 loci depicted a higher *H*_o_ than *H*_e_ on Zugspitze and Hoher Sonnblick, respectively. Mean comparisons of the ant species showed significant differences on Hoher Sonnblick when comparing multilocus heterozygosity (Mann–Whitney *U* test, *W* = 602, *p* = 0.003) but not when comparing *d*² (Mann–Whitney *U* test, *W* = 1256, *p* = 0.013; not significant after Bonferroni–Holm correction for multiple testing). The *F. aquilonia* exhibited a significantly lower multilocus heterozygosity mean (0.41) and a higher *d*² mean (54.05) than *F. exsecta* (0.51 and 29.90, respectively). Comparisons of locations within species yielded only one significant difference: multilocus heterozygosity within *F. exsecta* (Mann–Whitney *U* test, *W* = 636.5, *p* = 0.005), with Hoher Sonnblick depicting a significantly higher mean (0.51) than Zugspitze (0.43). No difference was found between *d* ^2^ values within *F. exsecta* (Mann–Whitney *U* test, *W* = 1195.5, *p* = 0.076) and the same held true for both Mann–Whitney *U* tests within *F. aquilonia* (*W* = 1629.5, *p* = 0.905 for multilocus heterozygosity and *W* = 1355, *p* = 0.152 for *d* ^2^). Inbreeding values were never significantly linked to fluctuating asymmetry of the head.

### PBT concentrations

**Figure 3:**
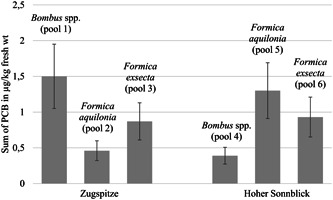
Sum of six polychlorinated biphenyl (PCB) indicators. Attention was focused on PCB 52 (2,2′,5,5′‐tetrachlorobiphenyl), PCB 101 (2,2′,4,5,5′‐pentachlorobiphenyl), PCB 138 (2,2′,3,4,4′,5′‐hexachlorobiphenyl), PCB 153 (2,2′,4,4′,5,5′‐hexachlorobiphenyl), and PCB 180. Concentrations are expressed in μg/kg of fresh weight (fresh wt) of pooled samples of *Bombus* spp. (Pool 1 and Pool 2) and *Formica aquilonia* and *Formica exsecta* from Zugpitze and Hoher Sonnblick (Pool 3–Pool 6). Detailed information on pooled samples is given in Table 1. The error bars indicate a measurement uncertainty of 30% (Eppe et al., 2015). Concentrations were determined by gas chromatography–high‐resolution mass spectrometry.

**Figure 4:**
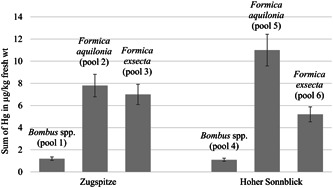
Sum of mercury (Hg). Concentrations were expressed in µg/kg fresh weight (fresh wt) of pooled samples of *Bombus* spp. (Pool 1 and Pool 2) and *Formica aquilonia* and *Formica exsecta* from Zugpitze and Hoher Sonnblick (Pool 3–Pool 6). Detailed information on pooled samples is given in Table 1. The error bars indicate a measurement uncertainty of 13% (Umweltbundesamt, 2015). Concentrations were determined via atomic fluorescence spectroscopy.

The sum of the six individually measured PCB concentrations (all PBTs were analyzed in pooled samples, consisting of several individuals; for details, see the *Materials and Methods* section and *Discussion* section) was between 0.39 and 1.5 µg/kg fresh weight in bumblebees (Figure 3). For *F. aquilonia*, the sum of PCBs was between 0.46 and 1.3 µg/kg fresh weight, and for *F. exsecta* it was between 0.87 and 0.93 µg/kg fresh weight. The Hg concentrations of the bumblebees were between 1.2 and 7.8 µg/kg fresh weight (Figure 4). In *F. aquilonia*, Hg concentrations between 7.8 and 11.0 µg/kg and in *F. exsecta* between 5.2 and 7.0 µg/kg were detected. In bumblebees, the values of Hg, PCB 138, PCB 153, and PCB 180 were higher on Zugspitze than on Hoher Sonnblick (Supporting Information, Table S8). In *F. aquilonia*, PCB 28, PCB 52, and PCB 101 concentrations were higher on Zugspitze than on Hoher Sonnblick. In *F. exsecta*, Hg, PCB 52, PCB 101, and PCB 180 concentrations were higher on Zugspitze than on Hoher Sonnblick.

### Correlation of PBT concentration with fluctuating asymmetry

In bumblebees, there was no significant link between the fluctuating asymmetry of hindwing and any PBT measurement. In contrast, the fluctuating asymmetry of the forewing was significantly linked to the concentrations of all individual PBTs. The fluctuating asymmetry of the ant heads was significantly linked to the concentrations of PCB 28, 52, 101, and 138, and partially to sampling locations. The fluctuating asymmetry of ant heads was never significantly linked to the concentrations of Hg.

## DISCUSSION

Examination of the effects of PBTs in wild insects is a young endeavor, to which we contribute insights toward a methodological framework. Our results indicate that the approaches chosen to face the major challenges in this field are appropriate.

First, PBT concentrations from wild samples are unknown a priori. For PBT detectability in the chemical analyses, individuals need to be pooled. Although a comparison of pooled insects decreases the statistical power, due to a decrease in sample number, we were able to detect body burdens above detection levels and to run statistical analyses. With the analyses of individual ant nests, comparisons of at least subpopulations were possible. The statistical evidence for a link of fluctuating asymmetry and PBT data from pooled individuals in bumblebees and ants from high‐elevation habitats is a step toward an established methodology for the examination of effects of PBTs on insects in field studies. Our results are supported by similar cases with a successful use of pooled samples to gain insights into PBT characteristics in insects (Liu et al., 2018). In contrast to laboratory studies on PBTs relying on specifically selected concentrations (Bueno et al., 2013; Mommaerts et al., 2011), field studies will thus be possible to capture potential effects caused by PBTs at concentrations occurring in the natural habitat of insects.

Fluctuating asymmetry is a measure of developmental instability, potentially caused by stressors such as environmental chemicals. Very strong fluctuating asymmetry levels can negatively affect insect populations, for example, via disadvantages in mating (Tadler et al., 1999) or even via increased mortality (Singha et al., 2007). Nevertheless, it must be assumed that levels of fluctuating asymmetry that reduce fitness of individuals to a large degree can rarely be detected in field‐sampled insects, because individuals thus affected die quickly due to predation or starvation (Gagliardi et al., 2016). Hence, it is also less likely that the respective concentrations of PBTs, which might be the reason for a high fluctuating asymmetry, will be sampled. We found that fluctuating asymmetry applies to bumblebee wings and ant heads from all locations, hinting at a potential developmental instability (Møller & Swaddle, 1997). Jenssen et al. (2010) confirmed the link between PCB concentrations and wing fluctuating asymmetry in vertebrates. In addition, in relevant studies of insects, correlations of the levels of other PBT concentrations, such as lead or OCPs, and fluctuating asymmetry could also be made (Antipin & Imasheva, 2001; Arambourou et al., 2012). Therefore, we cannot exclude the possibility that other PBTs triggered the detected fluctuating asymmetry in our study. However, our results confirm that fluctuating asymmetry may be a viable fitness proxy when one is examining the effects of PBTs on insects in the field in general.

The inbreeding levels found in our study were low. Quantifying the potential contributions of other stressors generally known to induce fluctuating asymmetry should improve the robustness of attributing phenotypical effects detected to PBTs in wild insects. For instance, fluctuating asymmetry may also occur due to developmental stochasticity or parasites (Polak, 2003). Other morphological stress symptoms like changes in size, mass of body parts, or entire organisms may be appropriate as well (Daugaard‐Petersen et al., 2018; Schindler, 1990; Van de Merve et al., 2010) and worth testing in a complementary study. Supplementary laboratory studies, exposing organisms to fixed PCB and Hg concentrations and comparing them with a control group, would be essential for obtaining experimental evidence of causality between chemical pollution and fluctuating asymmetry. In our study, Hg, exhibiting far higher concentrations than PCBs, could not be linked to fluctuating asymmetry in any model using ant data. This finding indicates that Hg does not cause fluctuating asymmetry at the concentrations detected in the ants’ bodies. However, in bumblebees Hg was linked to fluctuating asymmetry. Additional research would be useful to further investigate the links between fluctuating asymmetry and Hg in insects.

In addition to PBTs, endogenous population genetic factors such as inbreeding might influence fluctuating asymmetry (Băncilǎ et al., 2010). The results of the population‐genetic analyses confirm that the quality of markers used in the present study was adequate. Few bumblebee loci and many ant loci proved to be linked to or deviate from Hardy–Weinberg equilibrium, indicating the close relatedness of nestmates, which can cause linkage disequilibrium and deviations from Hardy–Weinberg equilibrium. However, in our study, *d* ^2^ and multilocus heterozygosity were never significantly linked to fluctuating asymmetry values. Thus, no evidence for a link between inbreeding and fluctuating asymmetry in any organism was found. A lack of interference makes sense, given that inbreeding does not seem to arise at high levels in these populations (Supporting Information, Figures [Link], [Link]) compared with other data of social Hymenoptera (Graur, 1985; Haag‐Liautard et al., 2009). The low portion of loci with higher *H*_o_ than *H*_e_ might suggest otherwise, but *H*_o_ is high, throughout, hinting at a high distribution of genetic variation and low inbreeding. However, degrees of fluctuating asymmetry do not necessarily increase with stronger inbreeding, as shown by Fowler and Whitlock (1994), who found no such link in moderately inbred *Drosophila melanogaster*. Additional research with highly inbred bumblebees and ants would be useful to demonstrate a potential effect of inbreeding on fluctuating asymmetry.

The shortcoming of the sampling was the low number of bumblebee specimens collected from Hoher Sonnblick. The data points of both sampling locations were grouped, and an influence of the location could not be deduced. The results from fluctuating asymmetry and microsatellite analyses of bumblebees from Hoher Sonnblick should be interpreted cautiously. The similar proportion of both bumblebee species found at Zugspitze was a good prerequisite for comparisons between both species from this location. The bumblebee activity is generally high in both locations, but the collection late in the season reduced our sample size. In addition, many of the active bumblebees in August and September are males that hatch at the end of the season. As aforementioned in the section *Chemical analysis of body burdens* of *Materials and Methods*, males as well as individuals from other bumblebee species were used to test whether bumblebees are overall suitable for body burden analyses. For an inbreeding analysis in the context of PBTs, bumblebees should be collected earlier in the year. Due to their frequent colonization in both study locations connected with high abundances in each nest, ants proved to be a suitable organism for the present study. The collection is easy, their nests guarantee consistent sampling for perennial studies, and sampling results are less subject to stochasticity. Both ant species are omnivore and forage for a long period during the year (Seifert, 2018). Given that PBTs bioaccumulate along food chains (Jones & de Voogt, 1999), the concentrations may generally be higher in ants than in bumblebees, which are herbivores (Goulson, 2003). However, geometric morphometrics are easier to carry out with bumblebees because the wings are easy to position for photography.

The measured concentrations of PCBs in *F. exsecta* were similar in both study areas. At Zugspitze, the concentrations of PCBs were higher in bumblebees than in *F. aquilonia*. In contrast, at Hoher Sonnblick, the concentrations of PCBs were lower in bumblebees than in *F. aquilonia*. This difference can be due to the different trophic levels of bumblebees and ants; as primary consumers, bumblebees can be affected to a higher degree by recently deposited PCBs on plant tissue, whereas ants as omnivores could be more representative of a long‐term contamination of the entire ecosystem. The differences in concentrations of these two taxa across locations could thus possibly represent differences in contamination histories across locations. The different concentrations of PCBs in *F. aquilonia* of the two study sites could be explained by the characteristics of the prevailing vegetation at the sampling sites. At Hoher Sonnblick, the nests sampled were in a more densely forested area than at Zugspitze. Nizzetto et al. (2006) showed a filter effect of forests for PCBs on mountains, with a significant enhancement of the deposition of PCBs and a more strongly polluted habitat than in less wooded areas. This filter effect of forests has also been demonstrated for Hg (Gworek et al., 2020). Thus, such filter effects may result in a slightly higher concentration in *F. aquilonia*, which colonize forested areas, than in *F. exsecta*, which live in less wooded habitat (Seifert, 2000).

In bumblebees, the concentrations of PCBs were analyzed in pooled samples comprising several species,because we did not sample sufficient individuals of any single species. Strictly taken, this implies that correlations between concentrations of PCBs and fluctuating asymmetry were not performed on the same sample sets. In further studies, this drawback should be avoided by sampling bumblebees earlier in the season to obtain more biomass ideally from a single species. In any case, it is useful in our view to sample, for example, both bumblebees and ants, and if possible, insects of additional trophic levels.

The quality of studies could be increased by including additional PBT data. De Voogt et al. (1990) recommend PCBs 105, 118, 126, and 156 to be integrated in every environmental monitoring due to their elevated toxicity. Including more PBTs in environmental monitoring may contribute to a deeper understanding of their effects on wild insects. However, when more PBTs are included, more biomass is necessary for chemical analysis. The investigation of other endpoints, such as body weight, reproduction, or behavior, in comparison with the concentration of PBTs in the bodies of wild Alpine insects would be a further extension of our present research. Links of these endpoints have already been found in other insects (Damiens et al., 2013; Fournier‐Level et al., 2016; Wu et al., 2021).

## CONCLUSIONS

We hope that our results will help to establish a methodology to examine PBT effects on wild insects in multiple ways. First, data from pooled individuals are a reliable solution to the problem of unpredictable or too low PBT concentrations in individuals, which should encourage further similar studies in insects. Second, fluctuating asymmetry may be a reliable proxy for overall PBT toxicity. To confirm a potential interference in the links of inbreeding and fluctuating asymmetry, this method may be repeated in highly inbred populations. This might be useful to optimize the methodology presented in our study. The same holds true for populations under heavier PBT load, because there are cases of much higher contaminations than in the locations that were examined in the present study (Zhou et al., 2016). Combining field and laboratory studies could also shed more light on PBT effects on fluctuating asymmetry in wild insects.

## Supporting Information

The Supporting Information is availableon the Wiley Online Library at https://doi.org/10.1002/etc.5303.

## Author Contributions Statement

**Veronika Rosa Hierlmeier**: Conceptualization; Methodology; Investigation; Data curation; Formal analysis; writing. **Nils Struck**: Investigation; Methodolgy; Formal analysis; writing. **Timotheus Kopf**: Conceptualization;. **Korbinian Peter Freier**: Conceptualization; Formal analysis; writing; Supervision; Project administration; Funding acquisition. **Florian Michael Steiner**: Conceptulization; Formal analysis; writing; Supervision; Project administration; Funding aquisition. **Birgit Christiane Schlick‐Steiner**: Conceptualization; Writing; Supervision; Project administration; Funding acquisition. **Anna Malena Hofinger**: Investigation; Formal analysis. **Victoria Leitner**: Investigation; Formal analysis. **Philipp Jakob Ernest Stromberger**: Investigation; Formal analysis. **Patrick Krapf**: Methodology; Formal analysis; Software; Writing.

## Supporting information

This article includes online‐only Supporting Information.

Supplementary information.

Supplementary information.

Supplementary information.

Supplementary information.

Supplementary information.

Supplementary information.

Supplementary information.

Supplementary information.

Supplementary information.

Supplementary information.

Supplementary information.

Supplementary information.

Supplementary information.

Supplementary information.

## Data Availability

Data, associated metadata, and calculation tools are available from the corresponding author (vhierlmeier@googlemail.com).
