# Supplementary figures and images for: Persistent, Bioaccumulative, and Toxic Chemicals in Wild Alpine Insects: A Methodological Case Study
Source: Environ Toxicol Chem. 2022 Mar 21;41(5):1215–27. doi: 10.1002/etc.5303 (PMC9311829; doi:10.1002/etc.5303)

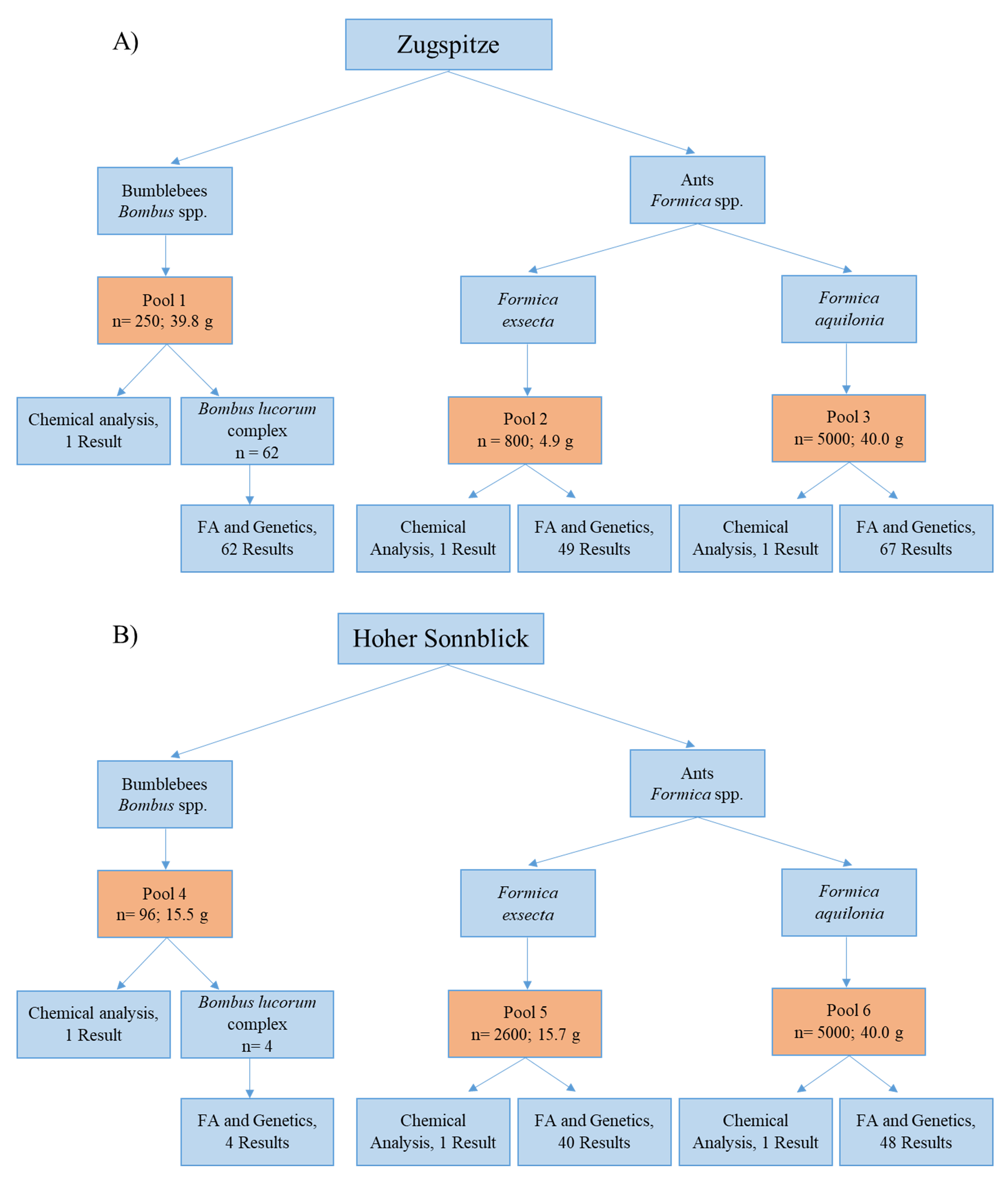

Supplement: Supplementary file 1 — Supplementary information. [file ETC-41-1215-s002.tif]

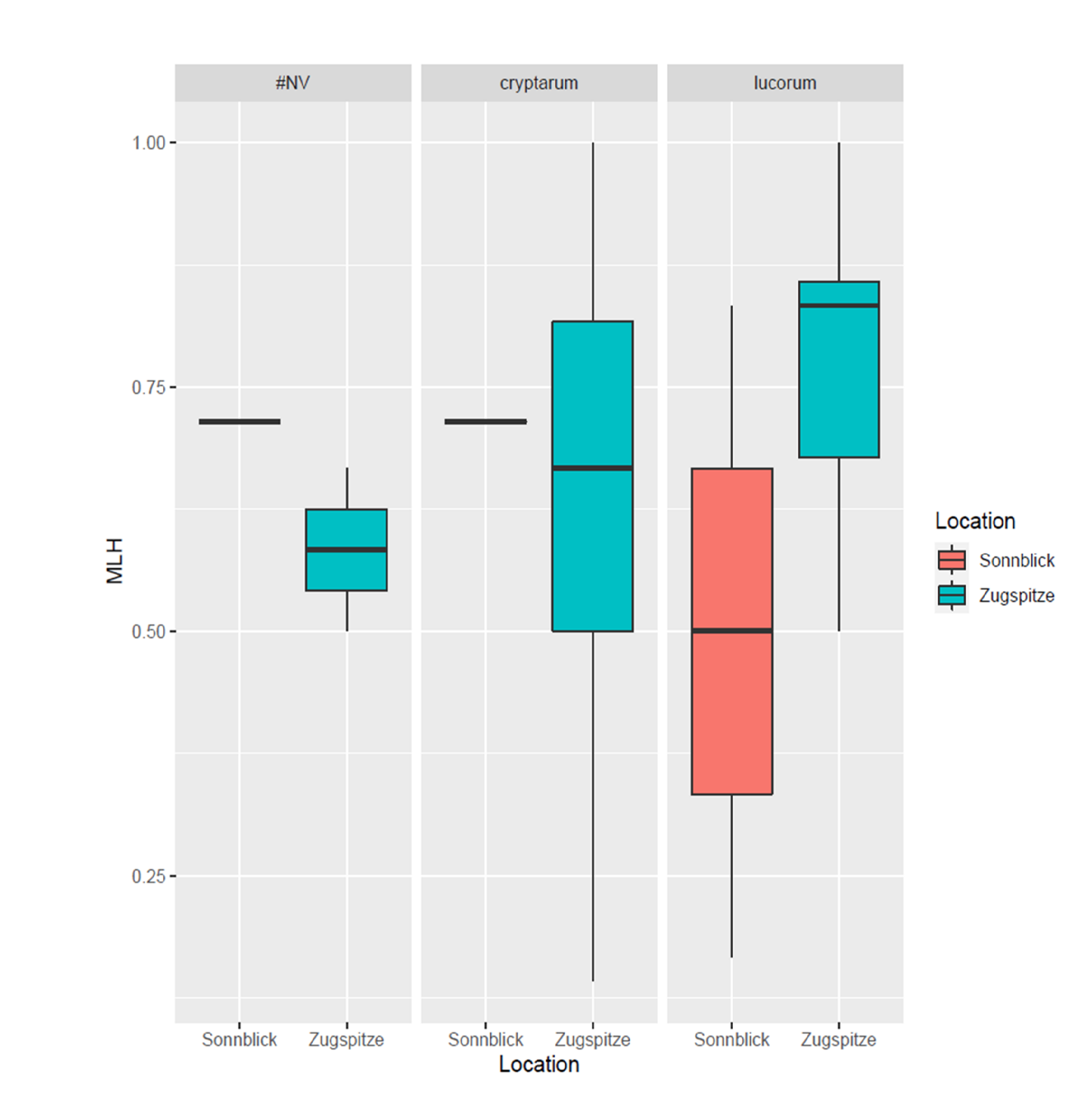

Supplement: Supplementary file 2 — Supplementary information. [file ETC-41-1215-s010.tif]

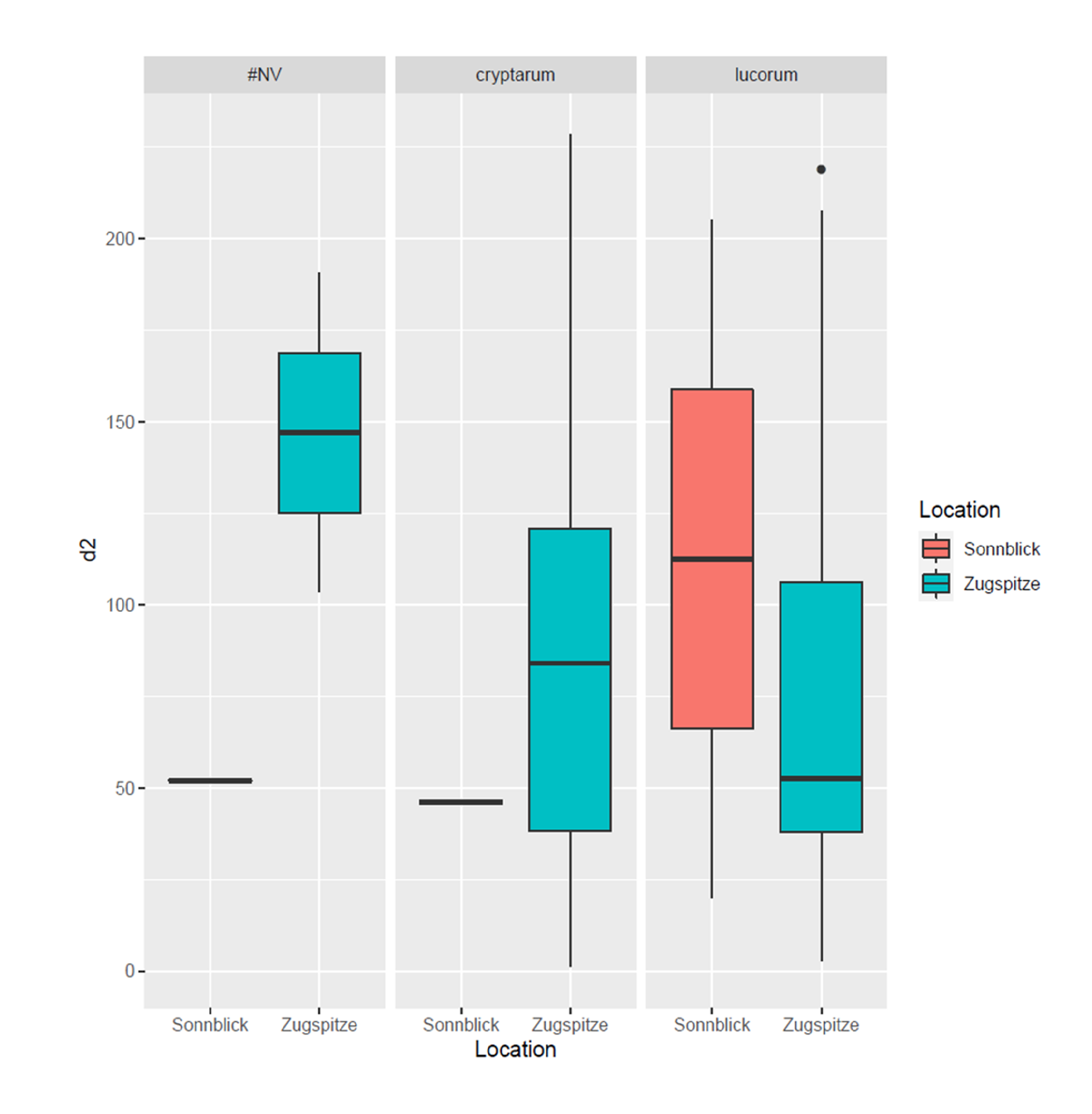

Supplement: Supplementary file 3 — Supplementary information. [file ETC-41-1215-s012.tif]

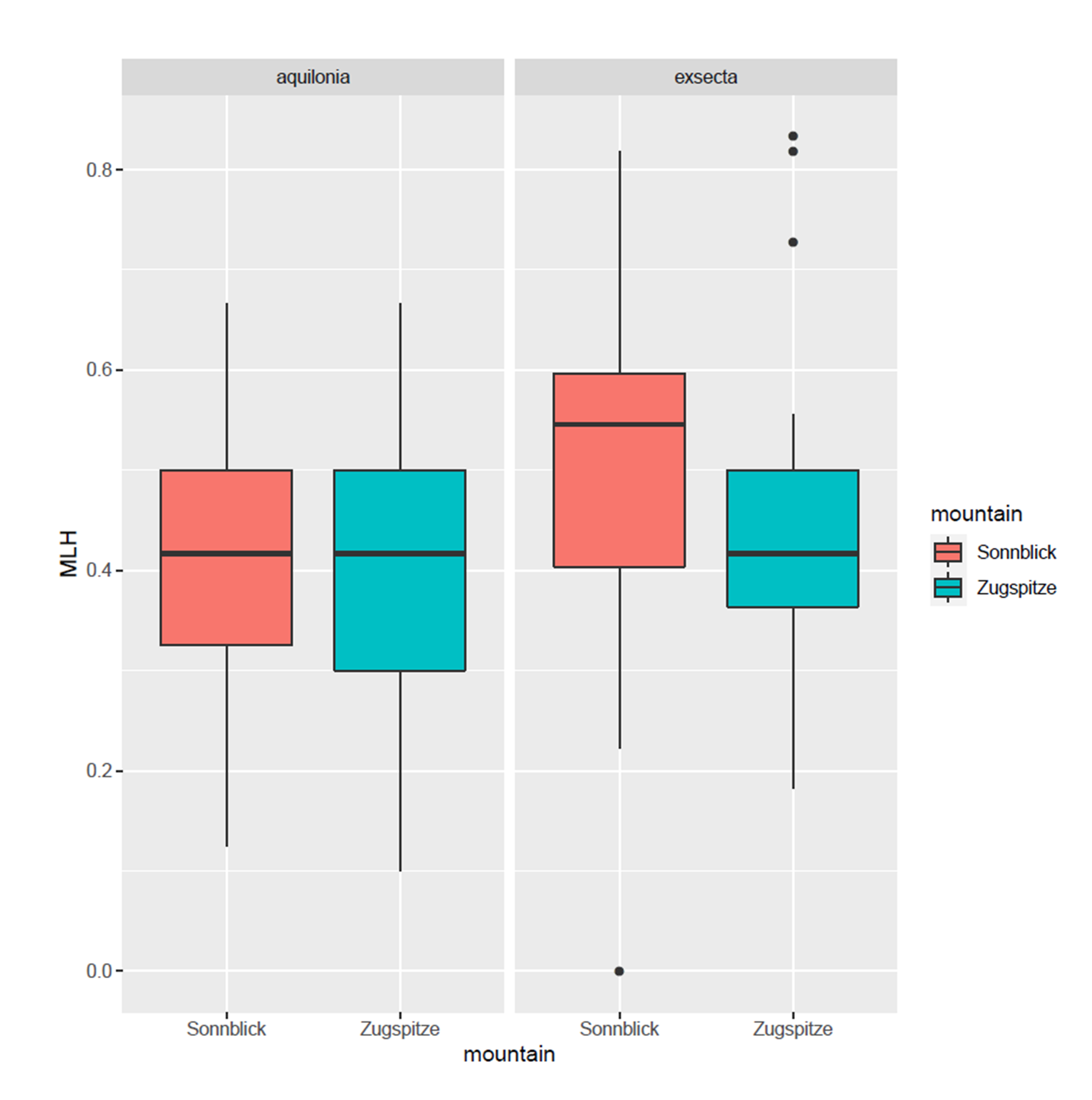

Supplement: Supplementary file 4 — Supplementary information. [file ETC-41-1215-s004.tif]

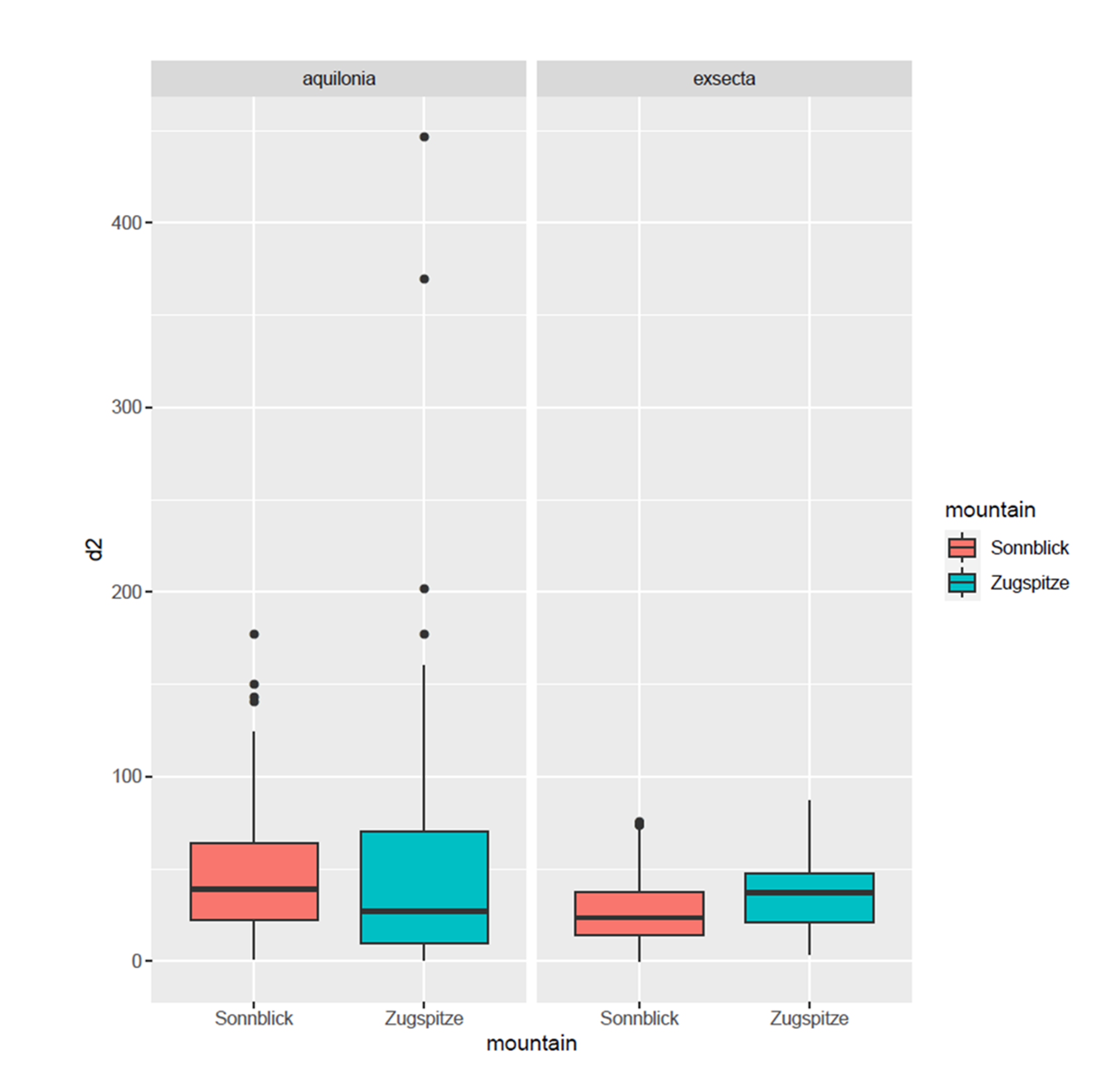

Supplement: Supplementary file 5 — Supplementary information. [file ETC-41-1215-s007.tif]
