# Supplementary material for: Persistent, Bioaccumulative, and Toxic Chemicals in Wild Alpine Insects: A Methodological Case Study
Source: Environ Toxicol Chem. 2022 Mar 21;41(5):1215–27. doi: 10.1002/etc.5303 (PMC9311829; doi:10.1002/etc.5303)
Supplement: Supplementary file 6 — Supplementary information. [file ETC-41-1215-s008.docx]

**Table S1.** Cleaning protocol for metal and glass utensils

| 1. | Rinse three to four times with toluene using a washing bottle |
| --- | --- |
| 2. | Rinse three to four times with acetone using a washing bottle |
| 3. | Evaporation under fume hood |
| 4. | Clean with brush in case of heavy dirtiness |
| 5. | Clean in dish washer (75 °C) using detergent |
| 6. | Thorough drying in a drying oven (105 °C) |
| 7. | Clean with isohexane Picograde^®^ (LGC Standards GmbH) using a washing bottle |
| 8. | Evaporation under fume hood |
| 9. | Heat in drying oven for 16-20 h (270 °C) |
| 10. | Cool down to room temperature |
