# Supplementary material for: Persistent, Bioaccumulative, and Toxic Chemicals in Wild Alpine Insects: A Methodological Case Study
Source: Environ Toxicol Chem. 2022 Mar 21;41(5):1215–27. doi: 10.1002/etc.5303 (PMC9311829; doi:10.1002/etc.5303)
Supplement: Supplementary file 7 — Supplementary information. [file ETC-41-1215-s006.docx]

**Table S2.** Primers and their respective polymerase chain reaction protocols used for cytochrome c oxidase I sequencing of bumblebees and ants.

| Primers | Organism | Initial denaturation | Cycles | Denaturation | Annealing | Extension | Final extension |
| --- | --- | --- | --- | --- | --- | --- | --- |
| No name | *Bombus* spp. | 93 °C,  1 min | 30 | 93 °C,  45 s | 45 °C,  1 min | 60 °C,  3 min | 60 °C,  4 min |
| LCO1490-HCO2198 | *Formica* spp. | 95 °C,  1 min | 35 | 95 °C,  1 min | 40 °C,  1 min | 72 °C,  90 s | 72 °C,  7 min |
