# Supplementary material for: Persistent, Bioaccumulative, and Toxic Chemicals in Wild Alpine Insects: A Methodological Case Study
Source: Environ Toxicol Chem. 2022 Mar 21;41(5):1215–27. doi: 10.1002/etc.5303 (PMC9311829; doi:10.1002/etc.5303)
Supplement: Supplementary file 8 — Supplementary information. [file ETC-41-1215-s003.docx]

**Table S3.** Detailed conditions of polymerase chain reactions for bumblebee and ant loci.

| Primers | Organism | Initial de-naturation | Cycles | De-naturation | Annealing | Extension | Final extension |
| --- | --- | --- | --- | --- | --- | --- | --- |
| 198,  327,  601 | *Bombus* spp. | 96 °C,  3 min | 35 | 96 °C,  45 s | 57 °C,  45 s | 72 °C,  45 s | 72 °C,  10 min |
| BT04, BT10, BT23, BL13 | *Bombus* spp. | 95 °C,  3 min | 39 | 92 °C,  30 s | 53 °C,  30 s | 72 °C,  30 s | 70 °C,  10 min |
| Fy3 | *Formica* spp. | 94 °C,  2 min | 39 | 94 °C,  30 s | 50 °C,  45 s | 72 °C  45 s | 72 °C,  10 min |
| FL21, FE7, FE1, FE13, FE15, FE38, FE42, FE49, FE51, P22 | *Formica* spp. | 94 °C,  2 min | 39 | 94 °C,  30 s | 56 °C,  45 s | 72 °C  45 s | 72 °C,  10 min |
| FE17 | *Formica* spp. | 94 °C,  2 min | 39 | 94 °C,  30 s | 60 °C,  45 s | 72 °C  45 s | 72 °C,  10 min |
| FE16, FE37 | *Formica* spp. | 94 °C,  2 min | 39 | 94 °C,  30 s | 65 °C,  45 s | 72 °C  45 s | 72 °C,  10 min |
