# Supplementary material for: Persistent, Bioaccumulative, and Toxic Chemicals in Wild Alpine Insects: A Methodological Case Study
Source: Environ Toxicol Chem. 2022 Mar 21;41(5):1215–27. doi: 10.1002/etc.5303 (PMC9311829; doi:10.1002/etc.5303)
Supplement: Supplementary file 9 — Supplementary information. [file ETC-41-1215-s014.docx]

**Table S4.** Primers with sequences, target organisms and references.

| Primer | Sequence (5’→3’) | Organism | Reference |
| --- | --- | --- | --- |
| 198 | F: AAATAGCTCGACACTGAGAGAC  R: ATCCATAAGCGTGTAAGAAAGT | *Bombus* spp. | Stolle et al., 2011 |
| 327 | F: AGAGAGAAGGAAGGACTCGT  R: CCGCAATTTACTTCTGATTATT | *Bombus* spp. | Stolle et al., 2011 |
| 601 | F: ACCGATTCGAAACTGATAGATA  R: TTCGAGGTAATTCGTTAAGTCT | *Bombus* spp. | Stolle et al., 2011 |
| BT04 | F: GAGAGAGATCGAATGGTGAGAGC R: TGAGCACGTTCTTTCGTTCAC | *Bombus* spp. | Reber Funk et al., 2006 |
| BT10 | F: TCTTGCTATCCACCACCCGC  R: GGACAGAAGCATAGACGCACCG | *Bombus* spp. | Reber Funk et al., 2006 |
| BT23 | F: GCAACAGAAAATCGTCGGTAGTG  R: GCGGCAATAAAGCAATCGG | *Bombus* spp. | Reber Funk et al., 2006 |
| BL13 | F: CGAATGTTGGGATTTTCGTG  R: GCGAGTACGTGTACGTGTTCTATG | *Bombus* spp. | Reber Funk et al., 2006 |
| Fy3 | F: ATTCATATGAGTTACATCGA  R: AGGTAATCAATATATTTAAG | *Formica* spp. | Hasegawa and Imai, 2004 |
| FL21 | F: GCCTGTCTCCTCTTCCGG  R: CATTGGTACCGTTCAACGATG | *Formica* spp. | Chapuisat, 1996 |
| FE7 | F: AAGCAATATCGCCAAAT  R: TTTCGGCTAACATACACA | *Formica* spp. | Gyllenstrand et al., 2002 |
| FE11 | F: TAACGGATTTTGCTCTAA  R: CGCTTAAACTTTGACAG | *Formica* spp. | Gyllenstrand et al., 2002 |
| FE13 | F: CTTCAAGCATATTTAAGCCA  R: ACGGAGATTAGGAACTGAAC | *Formica* spp. | Gyllenstrand et al., 2002 |
| FE15 | F: GCGAAAAAGCCACAGGA  R: ATCGATTTCGCGCTCC | *Formica* spp. | Gyllenstrand et al., 2002 |
| FE38 | F: ACGCCATCGTTTACCC  R: GATCGCACGTTCTGAGAG | *Formica* spp. | Gyllenstrand et al., 2002 |
| FE42 | F: CGGAGAACAACGATAATCTA  R: GGTGCTTTATTGAAATGTGT | *Formica* spp. | Gyllenstrand et al., 2002 |
| FE49 | F: AAAAATCAATAACTGAGCG  R: TTTACGATTTCGTTCTCC | *Formica* spp. | Gyllenstrand et al., 2002 |
| FE51 | F: TCGTCTCGCATCCACCACTA  R: GCGGGCGAAGAACAAAGG | *Formica* spp. | Gyllenstrand et al., 2002 |
| P22 | F: TTGCTGGAACACGTATCACAC  R: GTCGCATCGGATAGTGGGAG | *Formica* spp. | Trontti et al., 2003 |
| FE17 | F: CTCTCGATATCTCAATAAT  R: ACAGTCGTACATTCAATC | *Formica* spp. | Gyllenstrand et al., 2002 |
| FE16 | F: CCTGCGCCTGTAATCAGT  R: AAATCGCACGGCAAATAC | *Formica* spp. | Gyllenstrand et al., 2002 |
| FE37 | F: TTATTATTGCGGCATTACA  R: GGAGGACAGAAAGGAGAA | *Formica* spp. | Gyllenstrand et al., 2002 |
