# Supplementary material for: Persistent, Bioaccumulative, and Toxic Chemicals in Wild Alpine Insects: A Methodological Case Study
Source: Environ Toxicol Chem. 2022 Mar 21;41(5):1215–27. doi: 10.1002/etc.5303 (PMC9311829; doi:10.1002/etc.5303)
Supplement: Supplementary file 10 — Supplementary information. [file ETC-41-1215-s009.docx]

**Table S5.** Validation results for landmarking of bumblebee wings and ant heads to compare biological and technical variation.

| Organism | Effect | Sum of squares | Mean squares | df | F | p |
| --- | --- | --- | --- | --- | --- | --- |
| *Bombus* spp. | Individual | 5.74 x 10^-2^ | 8.39E-05 | 684 | 3.41 | <0.0001 |
|  | Fluctuating asymmetry | 1.68 x 10^-2^ | 2.46E-05 | 684 | 1.51 | <0.0001 |
|  | Imaging error | 2.35 x 10^-2^ | 1.63 x 10^-5^ | 1.44 x 10^3^ | 1.01 | 0.409 |
|  | Digitising error | 4.64 x10^-2^ | 1.61E-05 | 2.88 x 10^3^ |  |  |
| *Formica* spp. | Individual | 2.64 x10^-2^ | 2.72E-05 | 972 | 5.18 | <0.0001 |
|  | Nest | 2.05 x 10^-3^ | 3.79E-05 | 54 | 1.4 | 0.033 |
|  | Imaging error | 5.67 x 10^-3^ | 5.25E-06 | 1.08 x 10^3^ | 0.9 | 0.972 |
|  | Digitising error | 1.26 x 10^-2^ | 5.81E-06 | 2.16 x 10^3^ |  |  |

df = degrees of freedom
