# Supplementary material for: Persistent, Bioaccumulative, and Toxic Chemicals in Wild Alpine Insects: A Methodological Case Study
Source: Environ Toxicol Chem. 2022 Mar 21;41(5):1215–27. doi: 10.1002/etc.5303 (PMC9311829; doi:10.1002/etc.5303)
Supplement: Supplementary file 11 — Supplementary information. [file ETC-41-1215-s005.docx]

**Table S6.** Expected versus observed heterozygosity (H_e_/H_o_) in bumblebees.

|  | H_e_/H_o_ | |
| --- | --- | --- |
| Locus | *Bombus cryptarum* | *Bombus lucorum* |
| 601 | 0.57/0.48^b^ | 0.68/0.74^a^ |
| 198 | 0.79/0.58 | 0.75/0.84^a^ |
| 327 | 0.46/0.35 | 0.72/0.61 |
| BT04 | 0.45/0.29^b^ | 0.50/0.19^b^ |
| BT23 | 0.76/0.77^a b^ | 0.89/0.84 |
| BT10 | 0.76/0.68 | 0.90/0.84 |
| BL13 | 0.70/0.55^b^ | 0.75/0.74 |

^a^ higher H_o_ than H_e_

^b^ significant deviations from the Hardy-Weinberg equilibrium (HWE), p < 0.5
