# Supplementary material for: Persistent, Bioaccumulative, and Toxic Chemicals in Wild Alpine Insects: A Methodological Case Study
Source: Environ Toxicol Chem. 2022 Mar 21;41(5):1215–27. doi: 10.1002/etc.5303 (PMC9311829; doi:10.1002/etc.5303)
Supplement: Supplementary file 12 — Supplementary information. [file ETC-41-1215-s013.docx]

**Table S7.** Expected versus observed heterozygosity (H_e_/H_o_) in ants.

|  | H_e_/H_o_ | |
| --- | --- | --- |
| Locus | *Formica aquilonia* | *Formica exsecta* |
| FL21 | 0.68/0.46^b c^ | 0.67/0.76^a c^ |
| FE7 | 0.58/0.46^b c^ | 0.72/0.90^a c^ |
| FE11 | 0.64/0.44^b c^ | n.a. |
| FE37 | 0.64/0.44^b c^ | 0.31/0.38^a^ |
| FE15 | 0.00/0.00 | 0.29/0.30 ^a b^ |
| FE42 | 0.34/0.30 | 0.47/0.72^a c^ |
| FE16 | 0.79/0.50^b c^ | 0.84/0.32^b c^ |
| FE13 | 0.57/0.60^a b^ | 0.36/0.42^a^ |
| FE49 | 0.49/0.00^b c^ | 0.18/0.20^a b^ |
| P22 | 0.08/0.08^a^ | 0.55/0.54^b^ |
| FE17 | 0.33/0.30 | 0.53/0.08^b c^ |
| FE38 | 0.59/0.62^a b^ | 0.79/0.70^b c^ |
| FE51 | n.a. | 0.60/0.67^a c^ |
| Fy3 | 0.21/0.24^a^ | n.a. |

^a^ higher Ho than He

^b^ significant deviations from the Hardy-Weinberg equilibrium (HWE) on Zugspitze, p < 0.5

^c^ significant deviations from the Hardy-Weinberg equilibrium (HWE) on Hoher Sonnblick, p < 0.5

n.a. = not available
