# Supplementary material for: Persistent, Bioaccumulative, and Toxic Chemicals in Wild Alpine Insects: A Methodological Case Study
Source: Environ Toxicol Chem. 2022 Mar 21;41(5):1215–27. doi: 10.1002/etc.5303 (PMC9311829; doi:10.1002/etc.5303)
Supplement: Supplementary file 13 — Supplementary information. [file ETC-41-1215-s001.docx]

**Table S8.** Concentrations of PBTs in each pooled sample from both locations. Detailed informations according the sample code is given in Table 1A. The values of the concentrations are subject to a measurement uncertainty of ± 15% for Hg (Umweltbundesamt 2015) and 30% for PCBs (Eppe et al. 2015).

| Organism | Location | Sample code | Hg^a^ | PCB 28 ^b^ | PCB 52 ^b^ | PCB 101 ^b^ | PCB 138 ^b^ | PCB 153 ^b^ | PCB 180 ^b^ |
| --- | --- | --- | --- | --- | --- | --- | --- | --- | --- |
| *Bombus* spp. | Zugspitze | Pool 1 | 1.2 | 0.071 | 0.059 | 0.066 | 0.17 | 0.84 | 0.27 |
|  | Hoher  Sonnblick | Pool 4 | 1.1 | 0.12 | 0.11 | 0.086 | 0.009 | 0.042 | 0.027 |
| *Formica aquilonia* | Zugspitze | Pool 2 | 7.8 | 0.031 | 0.042 | 0.076 | 0.14 | 0.14 | 0.037 |
|  | Hoher Sonnblick | Pool 5 | 11 | 0.021 | 0.027 | 0.016 | 0.22 | 0.83 | 0.2 |
| *Formica*  *exsecta* | Zugspitze | Pool 3 | 7 | 0.16 | 0.099 | 0.13 | 0.1 | 0.27 | 0.12 |
|  | Hoher Sonnblick | Pool 6 | 5.2 | 0.17 | 0.089 | 0.076 | 0.12 | 0.4 | 0.081 |

^a^: values in µg*kg^-1^

^b^: values in ng*g^-1^
