# Supplementary material for: Persistent, Bioaccumulative, and Toxic Chemicals in Wild Alpine Insects: A Methodological Case Study
Source: Environ Toxicol Chem. 2022 Mar 21;41(5):1215–27. doi: 10.1002/etc.5303 (PMC9311829; doi:10.1002/etc.5303)
Supplement: Supplementary file 14 — Supplementary information. [file ETC-41-1215-s011.docx]

**SUPPLEMENTAL FIGURE CAPTIONS**

**Figure S1.** Overview of organisms sampled on Zugspitze (A) and Hoher Sonnblick (B). The number and biomass of organisms examined in the chemical analysis as a pooled sample, as well as the number of organisms analysed for fluctuating asymmetry (FA) and genetics are given.

**Figure S2.** Boxplots of multilocus heterozygosity in bumblebees (*B. cryptarum* and *B. lucorum*; #NV are unidentified *Bombus* spp. individuals) from Zugspitze (indicated in green) and Hoher Sonnblick (indicated in orange). Horizontal lines: mean values. Boxes: interquartile ranges. Vertical lines: ranges.

**Figure S3.** Boxplots of mean squared distance between alleles in bumblebees (*B. cryptarum* and *B. lucorum*; #NV are unidentified *Bombus* spp. individuals) from Zugspitze (indicated in green) and Hoher Sonnblick (indicated in orange). Horizontal lines: mean values. Boxes: interquartile ranges. Vertical lines: ranges. Dots: outliers.

**Figure S4.** Boxplots of multilocus heterozygosity in ants (*Formica aquilonia* and *Formica exsecta*) from Zugspitze (indicated in green) and Hoher Sonnblick (indicated in orange). Horizontal lines: mean values. Boxes: interquartile ranges. Vertical lines: ranges. Dots: outliers.

**Figure S5.** Boxplots of mean squared distance between alleles in ants (*Formica aquilonia* and *Formica exsecta*) from Zugspitze (indicated in green) and Hoher Sonnblick (indicated in orange). Horizontal lines: mean values. Boxes: interquartile ranges. Vertical lines: ranges. Dots: outliers.
